# Novel adjuvants in allergen-specific immunotherapy: where do we stand?

**DOI:** 10.3389/fimmu.2024.1348305

**Published:** 2024-02-23

**Authors:** Yen-Ju Lin, Jennifer Zimmermann, Stefan Schülke

**Affiliations:** ^1^Section Molecular Allergology, Paul-Ehrlich-Institut, Langen, Germany; ^2^Section Research Allergology (ALG 5), Division of Allergology, Paul-Ehrlich-Institut, Langen, Germany

**Keywords:** type I hypersensitivity, allergen-specific immunotherapy, adjuvant, CpG, virus-like particle, mannan, flagellin, Th1/Th2 responses

## Abstract

Type I hypersensitivity, or so-called type I allergy, is caused by Th2-mediated immune responses directed against otherwise harmless environmental antigens. Currently, allergen-specific immunotherapy (AIT) is the only disease-modifying treatment with the potential to re-establish clinical tolerance towards the corresponding allergen(s). However, conventional AIT has certain drawbacks, including long treatment durations, the risk of inducing allergic side effects, and the fact that allergens by themselves have a rather low immunogenicity. To improve AIT, adjuvants can be a powerful tool not only to increase the immunogenicity of co-applied allergens but also to induce the desired immune activation, such as promoting allergen-specific Th1- or regulatory responses. This review summarizes the knowledge on adjuvants currently approved for use in human AIT: aluminum hydroxide, calcium phosphate, microcrystalline tyrosine, and MPLA, as well as novel adjuvants that have been studied in recent years: oil-in-water emulsions, virus-like particles, viral components, carbohydrate-based adjuvants (QS-21, glucans, and mannan) and TLR-ligands (flagellin and CpG-ODN). The investigated adjuvants show distinct properties, such as prolonging allergen release at the injection site, inducing allergen-specific IgG production while also reducing IgE levels, as well as promoting differentiation and activation of different immune cells. In the future, better understanding of the immunological mechanisms underlying the effects of these adjuvants in clinical settings may help us to improve AIT.

## 1 Introduction

Allergic reactions are an increasing health and economic problem in developed countries (1–4). Type I allergies, the most common form of allergies, are caused by exaggerated immune responses directed against otherwise harmless antigens from our environment (so-called allergens). Immunologically, these immune responses are characterized by the induction of allergen-specific Th2 cells and IgE antibodies, which trigger IgE-dependent mast cell degranulation with the associated allergic symptoms.

Currently, the only treatment affecting the underlying disease with disease-modifying potential is allergen-specific immunotherapy (AIT). In this form of treatment, patients are confronted with increasing amounts of allergen(s) by either oral, subcutaneous (s.c.), or sublingual (s.l.) routes over long periods of time. If successful, AIT results in the re-establishment of clinical tolerance towards the offending allergen(s) and is associated with (I) a late decline in allergen-specific IgE, (II) an early increase in allergen-specific IgG subclasses, and (III) the induction of regulatory T- and B cell subsets (5).

One of the major obstacles in achieving efficient allergen-specific immune modulation is the fact that most pure allergen molecules only display a low degree of immunogenicity (6). Here, adding adjuvants to the low-immunogenic allergen(s) is a promising strategy to either enhance or modify the overall immune responses induced by the allergen(s).

Adjuvants increase the magnitude of immune responses directed against the co-applied antigen(s), allowing for both the application of lower antigen doses (which may reduce side effects) and the induction of robust immune responses against otherwise non/low-immunogenic antigens (7, 8). Furthermore, adjuvants can change the overall type of immune response as the co-applied antigens are taken up, processed, and presented in the context of the adjuvant-induced immune cell activation.

**Figure 1:**
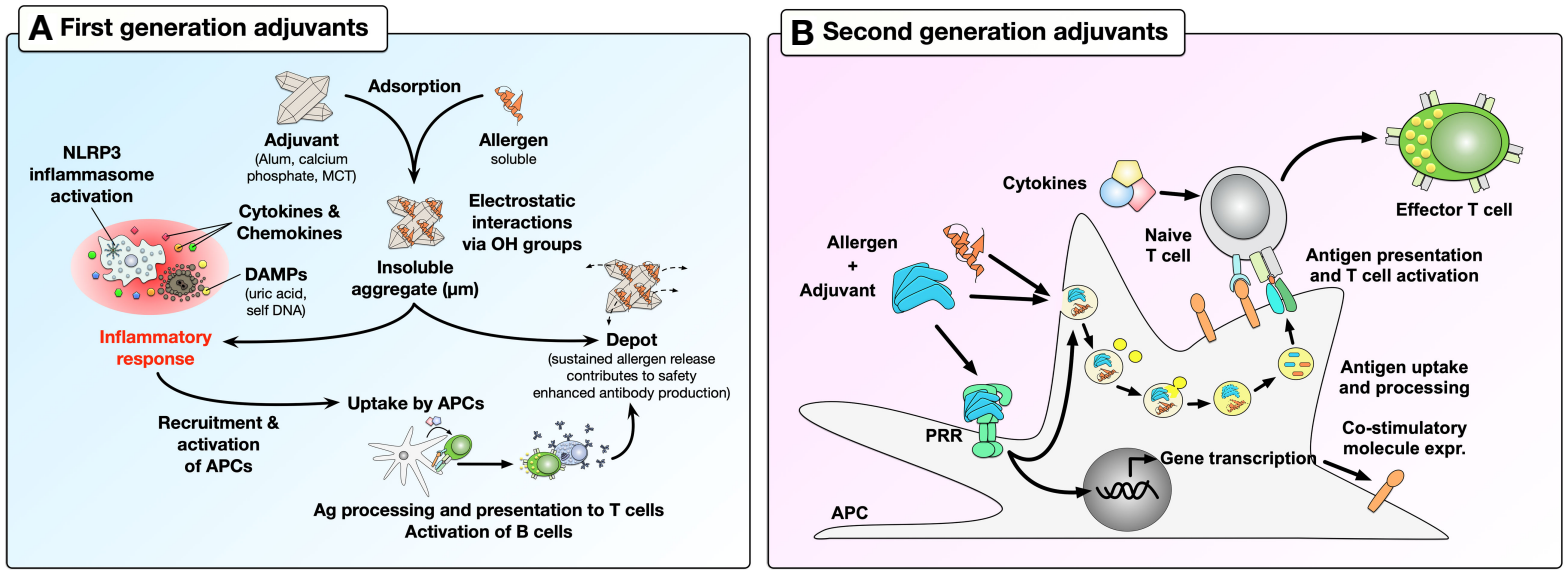
Boosting of immune responses by first- and second-generation adjuvants. First-generation adjuvants can adsorb soluble allergens through electrostatic interactions with the allergens hydroxyl groups to form insoluble aggregates **(A)**. Micron-sized adjuvant-allergen aggregates can (I) induce activation of immune cells, which further recruit and activate APCs, (II) be directly taken up by APCs, or (III) act as depots to effectively enhance allergen-specific antibody production. In contrast, second-generation adjuvants activate immune cells by triggering the activation of APCs via their PRRs **(B)**. Here, the binding of PAMPs (as second-generation adjuvants) to PRRs can induce transcriptional changes in APCs, leading to increased expression of co-stimulatory molecules and cytokine secretion. In addition, combining second-generation adjuvants with allergens can induce effective allergen uptake by APCs which then present allergen-derived peptides in the context of the adjuvant-induced APC activation. This results in effective T cell activation, thus triggering adaptive immune responses. For more information, see text. OH, hydroxyl group; MCT, microcrystalline tyrosine; NLRP3, NOD-, LRR- and pyrin domain-containing protein 3; PAMPs, pathogen-associated molecular patterns; DAMPs, damage-associated molecular patterns; APC, antigen-presenting cells; PRR, pathogen recognition receptor; Ag, antigen.

Depending on their mode of action, adjuvants can be further distinguished into first- and second-generation adjuvants (**Figure 1**). First-generation adjuvants used in AIT, such as aluminum salts, calcium phosphate, or microcrystalline tyrosine (MCT) serve as carriers, adsorbing antigens and forming insoluble, micron-sized adjuvant-antigen aggregates (9). The adjuvant-antigen aggregates activate immune cells, leading to the secretion of cytokines and chemokines as well as the formation of nucleotide oligomerization domain (NOD)-, leucine rich repeat (LRR)- and pyrin domain-containing protein 3 (NLRP3) inflammasomes. Subsequently, antigen-presenting cells (APCs) are recruited and activated by the production of strongly pro-inflammatory, bioactive IL-1β and IL-18, cell death, and the release of damage-associated molecular patterns (DAMPs) such as uric acid and self-DNA (**Figure 1**) (10–12). In addition, adjuvant-antigen aggregates can not only be directly taken up by APCs, but also act as depots, causing a slow release and effective presentation of the incorporated antigen(s) to APCs (**Figure 1**) (12). Despite the fact that first-generation adjuvants efficiently boost immune responses, especially antibody production, their application has a number of drawbacks for AIT. For example, aluminum hydroxide (alum)-based adjuvants may induce Th2 responses and IgE-production depending on the co-applied antigen, and lack the ability to induce CD8^+^ T cell responses (13, 14).

To improve first-generation adjuvants, recent studies have taken advantage of the discovery of pathogen recognition receptors (PRRs) (15). These receptors are abundantly expressed both on the surface and in the cytosolic compartments of APCs and other immune cells (B cells, T cells, neutrophils, mast cells, eosinophils, NK cells, innate-like lymphocytes), where they can promote immune responses by recognizing pathogen-associated molecular patterns (PAMPs) (15). Consequently, it was demonstrated that the use of different PAMPs (e.g., bacterial components, viral DNA/RNA) as second-generation adjuvants in combination with antigens could induce large-scale transcriptional changes in target cells, allowing them to alter their phenotype and function, e.g., by expressing co-inhibitory or co-stimulatory molecules, secreting cytokines, chemokines, and anti-microbial molecules; while also efficiently presenting antigens to T cells and thereby modulating T cell activation (**Figure 1**) (16).

**Figure 2:**
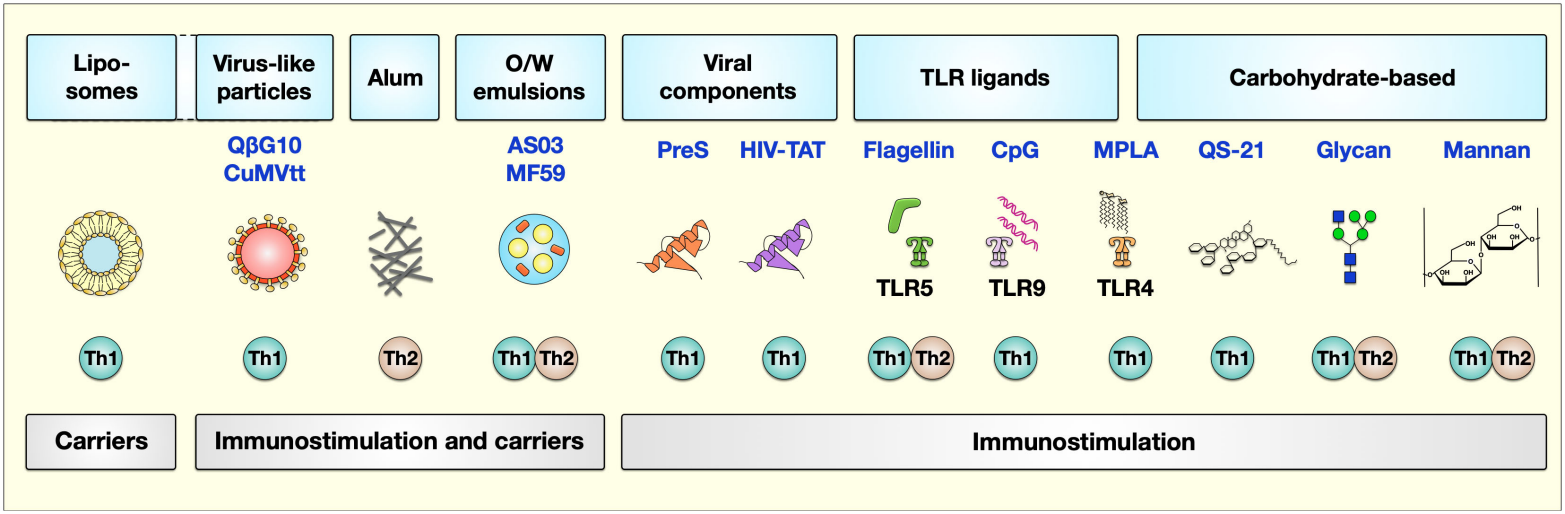
Adjuvants currently studied to improve AIT. The types of adjuvants can be classified based on their carrier or immunostimulatory capabilities. Here, liposomes serve as carriers, while virus-like particles, alum, and O/W emulsions have both carrier function and immune stimulatory characteristics. Besides viral components (the T-cell epitope derived from the hepatitis B virus PreS or the HIV-trans-activator of transcription (TAT)), TLR-ligands (flagellin, CpG, and MPLA), and carbohydrate-based adjuvants (QS-21, glycan, and mannan) have immunomodulatory properties triggering different kinds of immune responses. The described properties of these adjuvants to induce either Th1 or Th2 responses are indicated below the respective adjuvant. For more information, see text. AS, adjuvant system, alum: aluminum hydroxide; O/W, oil-in-water; Pres, HBV-derived T-cell epitope; TAT, HIV type 1 trans-activating regulatory protein; TLR, “Toll”-like receptor; MPLA, monophosphoryl lipid A. Figure modified after (17).

Currently, adjuvant research is a topic of great interest not only for improving allergy treatment but also for the development of other vaccines. In this review, we shortly summarize the adjuvants already in use in human AIT and subsequently focus on those adjuvants currently under investigation for further improving AIT (see also **Figure 2** for a general overview). The current state of clinical investigation of the discussed adjuvants is summarized in **Table 1**.

**Table 1:** Current state of clinical investigation of the discussed adjuvants.

Adjuvant/ Product name	Target	Most recent clinical study/ current state of clinical investigation	Year	Reference
MPLA/ Pollinex^®^ Quattro	Grass-, tree-, olive-, and weed allergy	Reduction of symptoms and increased production of allergen-specific IgG antibodies	2001	(18)
VLPs (QβG10)/CYT003	HDM- & ragweed allergy	Phase IIb study, CYT003 was well tolerated and safe, but there was no significant improvement compared to the placebo group, the study was prematurely terminated	2015	(19)
VLPs displaying Ara h 2	Peanut allergy	Phase I, on-going	2022	https://clinicaltrials.gov/study/NCT05476497
Viral components (PreS)/BM32	Grass pollen allergy	Phase II, patients had increased production of allergen-specific IgG_4_ without enhanced IgE responses	2019	(20)
Superfine dispersed β- (1,3)-glucan	Japanese cedar pollen allergy	Phase IV, patients showed reduction of clinical symptoms with lower pollen-specific IgE antibody levels	2007	(21)
Mannan	HDM allergy	Phase II, patients had no moderate or severe adverse reactions paralleled by induction of HDM-specific IgG_4_ antibodies	2022	(22)
Mannan	HDM- & birch pollen- allergy	Phase III, on-going	2022	NCT05400811, EudraCT: 2021-002252-36
CpG-ODN/TOLAMBA	Ragweed allergy	Phase IIb, no statistical significance between the treatment and placebo groups, termination of clinical development	2008	https://investors.dynavax.com/news-releases/news-release-details/dynavax-tolambatm-chamber-study-misses-primary-endpoint-company

MPLA, monophosphoryl lipid A; VLP, virus-like particle(s); HDM, house dust mite; PreS, T-cell epitope derived from the hepatitis B virus; CpG-ODN, CpG Oligodeoxynucleotides.

## 2 Adjuvants approved for human use in AIT

### 2.1 Aluminum hydroxide

Since Glenny et al. reported that alum could be used as an adjuvant in 1926 (23), it has become the most widely used adjuvant for different licensed human vaccines to prevent infectious diseases, such as diphtheria, tetanus, pertussis, hepatitis B, anthrax, and influenza (24). Alum adsorbs proteins via electrostatic interaction between alum and the proteins hydroxyl groups, thereby reducing allergen diffusion (lowering the chance of anaphylactic reactions) and prolonging the exposure of immune cells to these antigens at the injection site. Accordingly, alum has been included in most subcutaneous allergy immunotherapy (SCIT) products (25) in order to increase immune responses (12).

Recently, alum crystals were reported to also directly activate the NLRP3 inflammasome in mouse and human dendritic cells (DCs) and macrophages *in vitro*, resulting in secretion of the strongly pro-inflammatory and pyrogenic cytokines IL-1β and IL-18 [reviewed in (24)]. While mouse and human alum-stimulated DCs were shown to promote CD4^+^ T cell differentiation, especially towards Th2 cells via a prostaglandin E2-dependent mechanism [reviewed in (24, 26)], accumulated evidence from human application suggests alum to induce a more balanced Th1/Th2 immune profile depending on the formulation and the co-applied antigen (27, 28).

However, the use of alum is not without disadvantages. Alum frequently induces acute and chronic inflammation at the injection site, has a low biodegradability resulting in its accumulation upon repeated application (26, 29), and may have toxic effects; therefore, the application of alum as an adjuvant for type I allergy treatment is under ongoing investigation (30, 31).

### 2.2 Calcium phosphate

Calcium phosphate was first described as an adjuvant by Relyveld and colleagues in the 1960s (32–34). Although it is less commonly used than alum (35), it was used in AIT against seasonal rhinoconjunctivitis (36) as well as in vaccines against diphtheria, tetanus, pertussis, and poliomyelitis (37). Currently, because of reasons unknown to us, calcium phosphate adjuvanted AIT products are no longer used in the European market (Stallergenes Greer, personal communication).

Calcium phosphate is a natural component of the body with a better biodegradability and biocompatibility compared to alum, preventing its accumulation in the body upon repeated application (38, 39). While calcium phosphate displays a lower adjuvant activity than alum, it may still induce local adverse reactions, although of shorter duration (40). Upon subcutaneous injection, calcium phosphate induces active inflammation, involving the infiltration of neutrophils and macrophages (40). Earlier studies reported the induction of a similar IgG production in both animals and humans compared to alum (41, 42). However, the major advantage of calcium phosphate is its inability to induce the production of IgE in booster vaccinations (43) and immunotherapy (44). Furthermore, Wang et al. could show the induction of a cellular and humoral immune response as well as anti-cancer immunity upon the use of calcium phosphate-containing nanoparticles for vaccination purposes (45).

### 2.3 Microcrystalline tyrosine

MCT, the crystalline form of the non-essential amino acid L-tyrosine (46), is used as an adjuvant for AIT (47). Although MCT is less commonly used than alum (35), MCT has been shown to be both safe and effective for use in humans (46). It is a biodegradable depot adjuvant with a short half-life of 48 h (48), used in ultra-short course AIT for seasonal rhinitis (49, 50). The depot effect results in the slow release of the respective allergen(s), thereby prolonging their exposure to the immune system at the injection site (51).

Co-application of MCT with ovalbumin (OVA), a low immunogenic antigen, induces a B cell response leading to the production of antigen-specific IgG_1_, IgG_2a_, IgG_2b_, and IgG_3_ antibodies in mice that is comparable to alum, while inducing lower levels of both IgE and IL-4 (47). It induces lower levels of Th2 polarization compared to alum, which facilitates its use in AIT (52). Despite its more Th1-promoting adjuvant effects, MCT may induce both immediate and late local adverse reactions in patients undergoing AIT (53) and causes fewer, but still detectable, anaphylactic reactions in mice compared to alum (47). As was reported for alum, MCT was shown to induce caspase-dependent IL-1ß secretion *in vitro*, whereas the activation of the inflammasome was not essential for MCT-induced T or B cell response *in vivo* (47). In contrast to the TLR4-ligand monophosphoryl lipid A (MPLA) (see below), the promotion of T and B cell responses by either MCT or alum is independent of Toll-like receptor (TLR) signaling (47).

In AIT, MCT-adjuvanted native allergens or modified allergens (allergoids) are frequently used in combination with the TLR4-agonist MPLA, e.g. in the allergen therapeutic Pollinex^®^ Quattro (PQ, Bencard Allergy GmbH, Munich, Germany) for the treatment of seasonal allergic rhinoconjunctivitis (49, 54). The combination of MCT as a Th1 promoting, first-generation adjuvant and MPLA as a Th1-promoting second-generation adjuvant in AIT has promoted dose reductions (55). Since MCT boosts T-cell mediated responses (47), its use in vaccination against different infectious diseases is currently investigated (56–58). Here, MCT-adjuvanted nanoparticles induced enhanced B and T cell responses in pre-clinical models of malaria (57, 58) and cancer (59).

### 2.4 Monophosphoryl lipid A

MPLA is a carbohydrate-based adjuvant that activates TLR4 (60). It was developed by removing phosphate and fatty acid groups from the lipopolysaccharide (LPS) of *Salmonella minnesota* R595 by a series of organic extractions followed by mild acid and alkaline treatments (61). Although the TLR4-ligand LPS efficiently promotes immune responses when combined with antigens, its applicability as an adjuvant is strongly limited by its high toxicity (62). In contrast, MPLA maintains the adjuvant effect observed for LPS combined with a lower toxicity on human CD4^+^ T cells *in vitro* (63), and did not cause dangerous adverse effects in a human clinical trial (64). Therefore, several vaccines containing MPLA have either been tested in clinical trials or are already approved for human use like Fendrix^®^ (vaccination against hepatitis B virus), Cervarix^®^ (human papillomavirus-16 and -18), RTS,S^®^ (Malaria), and Pollinex^®^ Quattro (grass pollen allergy, in Germany only marketable according to therapy allergen ordinance but no marketing authorization) (50, 65–67). The overall immunological effects of MPLA are relatively well studied. It is known that MPLA can induce Th1-biased immune responses and promote both IgG_1_- and IgG_4_-dominated humoral immune responses without boosting IgE production (68, 69) and without the capacity to directly activate murine or human mast cells (70), making MPLA an attractive adjuvant for improving AIT. *In vitro*, MPLA was also shown to induce a pronounced, c-Jun N-terminal kinase mitogen-activated protein kinase (JNK-MAPK)- and mammalian target of rapamycin (mTOR)-dependent activation of glucose metabolism, IL-10 secretion, and CCL2 production in mDCs (71).

PQ is an allergen therapeutic containing glutaraldehyde-crosslinked pollen allergoids adsorbed to MCT that are further adjuvanted with MPLA (72). PQ was first developed in the 1970s for the treatment of grass pollen allergy, followed by the Pollinex-R, which included ragweed allergens (72). PQ was launched on the market in 1999 as named patient products for the treatment of allergic rhinitis (AR) caused by grass-, tree-, olive-, and weed allergens but so far has not received marketing authorization in Germany (72). Compared to traditional AIT approaches that normally require months to years of treatment, PQ is based on a “short-term specific immunotherapy (ST-SIT)” consisting of only four pre-seasonal injections with increasing allergen dosages (18). Clinical studies showed, that PQ treatment significantly reduced nasal and ocular clinical symptoms, while also shifting immune responses from allergic Th2- toward Th1-biased responses characterized by higher grass-pollen-specific IgG antibody production (18). Moreover, pre-clinical *in vivo* studies in rats and dogs showed no toxicological findings, and no significant local and systemic adverse events were reported in human clinical trials (18, 73).

Our own work demonstrated that both PQ and two commercial vaccines adjuvanted with MPLA could activate mDC metabolism (74). Activation of mDCs by PQ was mediated by a pronounced mTOR- and JNK-MAPK-dependent activation of glucose metabolism that regulated mDC-derived cytokine secretion (74). Finally, mDC glucose metabolism was also critical for the (Th1-biased) T cell priming capacity of PQ-stimulated mDCs (74).

Taken together, the available data for PQ show that combining allergen(s) with the adjuvants MPLA and MCT may improve major disadvantages of traditional AIT regarding long treatment periods, low patient adherence, and occurrence of side effects. However, so far, sufficient clinical human data fully supporting marketing authorization in Germany have not been reported. Moreover, as of January 2024, PQ has not been approved for marketing in any other European state (Bencard, personal communication).

## 3 Adjuvants currently investigated for use in human AIT

### 3.1 Liposomes

Liposomes are biodegradable nanoparticles (see (75) for a review of nanoparticles in AIT) that have been considered as adjuvants combining immune cell activation with effective packaging and delivering water-soluble antigens to target cells (76). Despite their potent immune-stimulatory capacity, liposomes may also cause severe side effects such as toxicity to cells of the mononuclear phagocyte system (monocytes, macrophages, and DCs), reducing the secretion of immune effector molecules by these cells, complement activation-related pseudoallergy, and organ damage to liver and spleen upon intravenous (i.v.) application [reviewed in (77–79)].

The potential of liposomes to carry allergens was first tested in 1991 by Audera’s group (80). In their experiments, house dust mite (HDM)- (*Dermatophagoides pteronyssinus*), grass- (*Lolium perenne*, *Phleum pratense*, *Parietaria judaica*, and *Artemisia vulgaris*), and cat dander-allergen extracts with or without liposome encapsulation were injected into BALB/c mice (80). Their results showed that liposome-encapsulated allergen extracts could induce higher levels of allergen-specific IgG paralleled by a lower IgE production (80). One disadvantage when using liposomes is their lack of inherent immune-activating capacity, which can be overcome by combining liposomes with additional adjuvants. For this, one liposome-based formulation, the commercial adjuvant system 01 (AS01), which additionally contains the TLR4-ligand MPLA (see above) and the natural saponin product QS-21 (see below) has already been approved for use in malaria- (Mosquirix^TM^) and shingles (Shingrix^(R)^) vaccines (81). Besides, a recent *in vivo* study also encapsulated CpG oligodeoxynucleotides (CpG-ODN), a synthetic TLR9-agonist, together with OVA in liposomes to evaluate their potential for allergy treatment (82). Their results showed that OVA encapsulated in liposomes could significantly reduce cutaneous anaphylactic reactions in an *in vivo* OVA-induced asthma mouse model (82). Moreover, liposomes encapsulating OVA and CpG-ODN, but not the OVA plus CpG-ODN mixture alone, reversed OVA-induced allergic lung inflammation in a mouse model (82). The inflammation-suppressing effect of the liposomes was shown to be myeloid differentiation primary response protein 88 (MyD88)-dependent, and mediated by CD11c^+^ DCs (82). Therefore, liposomes may have potential as carriers that encapsulate allergens and other adjuvants to improve allergy treatment.

### 3.2 Oil-in-water emulsions

Besides alum and liposome-based AS01, two oil-in-water (O/W) emulsion adjuvants AS03 (containing squalene, alpha-tocopherol, and polysorbate 80) and MF59 (consisting of squalene and the non-toxic emulsifiers Tween 20 and Span 85), have also been licensed for clinical use, especially for various influenza vaccines (83). Mechanistically, O/W emulsions have the advantage of gradually releasing the combined antigen at the injection site, which reduces the chance of anaphylactic reactions while at the same time stimulating the activation of plasma cells producing antigen-specific antibodies and generating mixed Th1/Th2 responses (84, 85). Moreover, MF59 was also shown to induce DC recruitment and increase their antigen uptake activity *in vivo* (86). Potential safety risks concerning AS03 were raised after the use of the influenza vaccine Pandemrix^(R)^ (adjuvanted with AS03) during the global indluenza A H1N1 pandemic in 2009. Pandemrix^(R)^ was later found to be associated with increased frequencies of narcolepsy in patients with an HLA-DQB1*0602 haplotype (reviewed in (87)). Mechanistically, the vaccine was suggested to increase the frequency of antibodies to the hypocretin (HCRT) receptor 2 that both cause sleep dysregulation via a loss of HCRT-producing neurons and cross-react with a particular fragment of influenza nucleoprotein (reviewed in (87)).

Currently, there are limited studies regarding the use of O/W as adjuvants to improve AIT. Recently, O’Konek and Baker, Jr. described nanoscale O/W emulsions (NE), which induced potent Th1- and Th17-polarized immune responses (88). *In vivo*, treatment of mice with NE-formulated peanut allergens (PN-NE) suppressed allergic responses after both oral or systemic peanut allergen challenge (88). Furthermore, a decreased production of the Th2 cytokines IL-4 and IL-13, as well as a higher production of the Th1 cytokine IFN-γ and the anti-inflammatory cytokine IL-10, were observed in PN-NE-treated & peanut-allergic mice (88).

### 3.3 Virus-like particles and viral components

**Figure 3:**
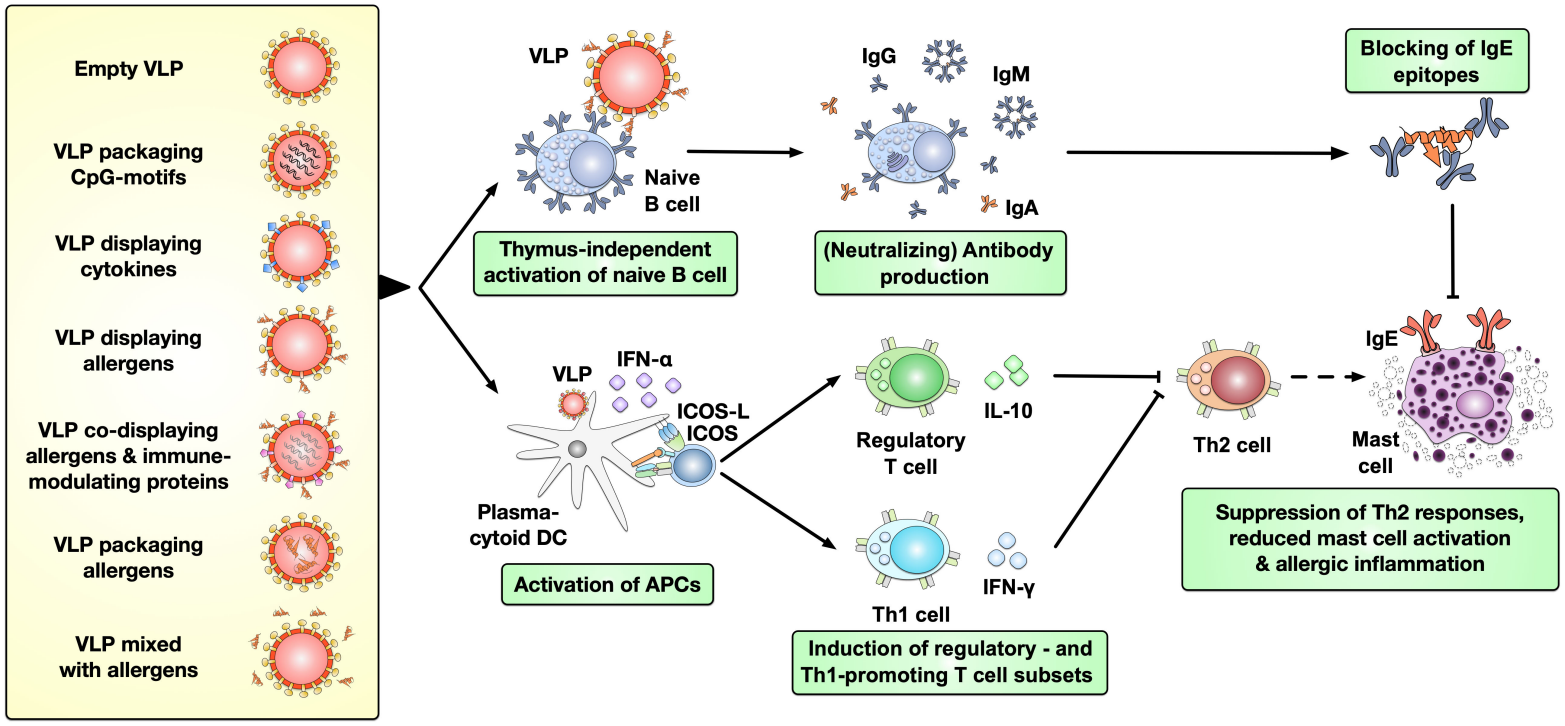
Types of VLPs investigated pre-clinically in AIT and their immunological effects. VLPs have been applied in pre-clinical studies to improve the treatment of allergies either as empty particles, packaging immune-stimulatory CpG-motifs, displaying either cytokines or allergen(s) on their surface, co-displaying both allergen(s) and immune-modulating proteins on their surface, packaging allergen(s) inside the VLPs, or mixed with allergen(s). VLPs were shown to thymus-independently activate naïve B cells, resulting in production of potentially neutralizing antibodies while also promoting the activation of plasmacytoid DCs (pDCs) (92–94). These APCs in turn favor the induction of regulatory- and Th1 cells which suppress the allergy-causing Th2 cells. Together these effects of VLPs may result in reduced mast cell activation and allergic inflammation [reviewed in (90)]. (p)DC, (plasmacytoid) dendritic cell; VLP, virus-like particle; IgG/A/E, immunoglobulin G/A/E.

Virus-like particles (VLPs) are multimeric entities that have the morphology of a native virus but do not contain viral genomic material and are therefore unable to replicate *in vivo*. Consequently, VLP-based vaccines have shown an improved safety profile compared to either live-attenuated or inactivated vaccines (89). They can serve as effective delivery carriers while offering strong immune-modulating capacity due to their highly repetitive and ordered structure (90). Several VLPs have already been licensed for clinical use: Cervarix^®^, Gardasil^®^, and Gardasil9^®^ for the prevention of human papillomavirus (HPV) infection, Hecolin^®^ for the prevention of hepatitis E, Recombivax HB^®^ and Sci-B-Vac^™^ for hepatitis B, and Mosquirix^™^ for malaria (90, 91). VLPs have also been studied for allergy treatment and the current strategies for using VLPs are reviewed in other publications (90). In brief, VLPs can be applied (I) alone, (II) packaging immune-stimulatory CpG-motifs, (III) display either cytokines or (IV) allergen(s) on their surface, (V) co-display both allergen(s) and immune-modulating proteins on their surface, (VI) package allergen(s) inside the VLPs, or (VII) mixed with allergen(s) to improve AIT (**Figure 3**) (90). Up to now, several studies applying different VLPs (except for VLPs displaying transforming growth factor beta 1 (TGF-β1) (95)) showed potential to improve allergic responses in allergic animal models *in vivo*, including reduced mast cell activation, suppressed Th2- while promoting Th1 responses, and upregulation of neutralizing antibody production [**Figure 3** and reviewed in (90)].

The largest number of clinical studies on VLPs in the field of allergology have been performed on VLPs derived from the bacteriophage Q beta (QβG10, all financed by Cytos Biotechnology AG, Schlieren, Switzerland). These form particles of approximately 30 nm in size that were used to encapsulate approximately 60 molecules of immune-stimulating A-type CpG ODN (96). In contrast to B-type CpGs ODNs, A-type CpG ODNs were described to be less stable and induce the secretion of interferon alpha (IFN-α) from plasmacytoid DCs (pDCs) rather than IL-12 (96).

In an open-label phase I pilot trial 20 patients with HDM allergy were treated for 10 weeks with a mixture of QβG10 (termed CYT003) and an alum-adsorbed HDM extract (96). Here, the combination of QβG10, alum and allergens resulted in a 10-fold reduction in self-reported symptoms while at the same time increasing HDM-specific IgG_1_, IgG_2_, and IgG_4_ antibody production compared to before the treatment (96). These observations encouraged larger placebo-controlled trials, the first of which was published in 2011 by Klimek and coauthors. In a double-blind, randomized, placebo-controlled clinical trial enrolling 299 patients with HDM allergy, patients were treated for two months with either 0.5 or 1 mg of QβG10 alone. The authors reported QβG10 to dose-dependently reduce the average combined symptom medication score, while the dose of 1 mg QβG10 also resulted in a higher number of patients with increased allergen tolerance (97). Therefore, the authors suggested QβG10 to be an “allergen-independent treatment with favorable safety profile and therapeutic treatment benefit within 2 months” (97). In the second reported parallel-group, double-blind, randomized trial, 63 asthmatic patients undergoing steroid withdrawal were treated with 0.9 mg QβG10 (without additional allergen) (98). Also in this patient collective, QβG10 was reported to reduce asthma-related symptom scores while increasing the number of patients with well-controlled asthma (98). However, in a final double-blind phase IIb study with 365 patients with not sufficiently controlled, persistent moderate-to-severe allergic asthma, Casale et al. reported both no significant difference between the QβG10 treatment and placebo groups at week 12 and no significant differences in secondary outcomes (19). Therefore, the study was prematurely terminated due to a lack of efficacy compared to patients receiving standard inhaled glucocorticosteroid therapy with or without long-acting beta-agonists (19). It was stated by the authors that “continued clinical development of CYT003 in this indication is unlikely, but these results should not preclude further exploration of immune-modifying therapies in allergic asthma” (19).

In a related approach, Storni et al. under participation of Allergy Therapeutics Ltd, (Worthing, UK), recently published data on optimized cucumber mosaic virus (CuMV)-derived VLPs displaying allergen(s) (99). These VLPs encapsulate immune-stimulatory *E. coli* RNA while at the same time co-displaying tetanus toxin-derived epitopes (as universal antigens inducing recall responses in formerly tetanus-vaccinated individuals) as well as either the major peanut allergens Ara h 1, Ara h 2, or roasted peanut extract (99). In BALB/c mice sensitized with peanut extract plus alum intraperitoneal (i.p.), vaccinated with CuMV-VLPs s.c., and challenged with peanut extract i.v., CuMV-VLP treatment strongly reduced the peanut-induced temperature drop (99). Here, CuMV-VLPs displaying either Ara h 1, Ara h 2, or roasted peanut extract showed a similar efficacy, suggesting that vaccination against a single allergen was able to protect against challenge with the whole extract (99). In mechanistical studies, the CuMV-VLPs displaying Ara h 1 were shown to mediate their protective effects via a strong induction of peanut-specific IgG2_a_ and IgG_2b_ antibodies that down-regulated mast cell activation via FcγRIIB-dependent inhibitory signaling (99).

Finally, a phase I clinical trial sponsored by Allergy Therapeutics Ltd was initiated in January 2022 to analyze VLPs that display the major peanut allergen Ara h 2 for treating peanut-allergic patients (https://clinicaltrials.gov/ct2/show/NCT05476497). This trial may provide more insights in the potential of VLPs for allergy treatment.

Besides VLPs, viral components which have immune-activating capacity have also been proposed as adjuvants for allergy treatment. For example, the hepatitis B virus (HBV)-derived PreS T-cell epitope fused with different allergens was tested both *in vivo* (in mice and rabbits) (100, 101) and in a human phase II clinical trial sponsored by Biomay AG (Vienna, Austria) (20, 102). Two non-allergenic rFel d 1 (the major cat allergen)-derived peptides fused with PreS strongly suppressed basophil activation while inducing production of Fel d 1-specific IgG antibodies after injection into either mice or rabbits (101). Moreover, a fusion protein consisting of major birch pollen allergen Bet v 1-derived peptides and PreS (rBetv1:PreS) also induced higher production of Bet v 1-specific IgG antibodies from rabbits (100). More detailed *in vitro* analyses demonstrated rBetv1:PreS to reduce T cell activation in PBMCs from allergic patients, which was paralleled by both higher anti-inflammatory IL-10- and Th1 cytokine IFN-γ production (100). Moreover, BM32, a vaccine candidate fusing PreS with hypoallergenic peptides derived from several major timothy grass pollen allergens adsorbed to alum induced allergen-specific IgG_4_ responses in a two-year AIT approach (phase II study, sponsored by Biomay AG) (20, 102). BM32 also demonstrated a good safety profile with no reported anaphylactic reactions and T cell-mediated side effects while reducing both IgE reactivity and allergen-specific Th2 cytokine secretion during the two years of treatment (20).

In addition to PreS, there are also two more pre-clinical mouse studies that generated fusion proteins combining viral proteins and allergen-derived peptides: Salari et al. used the HIV type 1 (HIV-1) trans-activating regulatory protein (TAT) fused with *Chenopodium album* pollen allergen Che a 3 (rTAT-Che a 3) (103), and Edlmayr et al. used rhinovirus viral protein 1 (VP1) fused with the major grass pollen allergen Phl p 1 peptide P5 (rVP1-P5) (104). Both approaches demonstrated the induction of higher allergen-specific IgG antibody production in mice by the respective fusion proteins (103, 104). Moreover, rTAT-Che a 3 was shown to enhance T_reg_-mediated immune responses, including lower production of the Th2 cytokine IL-4 as well as enhanced secretion of the Th1 cytokine IFN-γ (103). On the other hand, rVP1-P5 induced grass pollen-specific Th1-biased immune responses with reduced Th2 activation were observed in *in vitro* splenocyte cultures (104). Both studies were pre-clinical not reporting potential side effects, but Edlmayr and colleagues reported rVP1-P5 to not react with IgE antibodies from grass pollen allergic patients, to lack allergenic activity upon contact with basophils from allergic patients, and to induce protective IgG antibodies in mice or rabbits that blocked both IgE reactivity to Phl p 1 and Phl p 1-induced basophil degranulation (104).

In summary, these findings show that VPLs and certain viral proteins may be used as adjuvants for generating allergy vaccines.

### 3.4 Carbohydrate-based adjuvants

Carbohydrates are the most common type of biomolecule that can be found in nature. Accordingly, they have an important role in modulating both innate and adaptive immune responses (105). Carbohydrates combine a high biocompatibility with low toxicity, making them interesting novel adjuvant candidates. Currently, two carbohydrate-based adjuvants, QS-21 and MPLA (already discussed above), have already been licensed for clinical use. Moreover, several different other compounds have been investigated. The current state of knowledge is briefly summarized below.

#### 3.4.1 Saponin-based adjuvants (QS-21)

QS-21 is a triterpene glycoside that can be purified from the soap bark tree (*Quillaja saponaria*) (106). It has been tested as a therapeutic vaccine adjuvant in clinical trials against cancer (lymphoma, leukemia, melanoma, breast-, prostate-, ovary-, or lung cancer) and infectious diseases (HIV-1 and hepatitis B) (81, 107).

The mechanisms underlying the adjuvant effects of QS-21 are not extensively studied. We know that induction of immune responses by QS-21 is mediated by a pronounced activation of innate immune cells: upon injection, QS-21 activates subcapsular CD11b^+^CD169^+^ macrophages in draining lymph nodes that in turn recruit neutrophils and DCs (108, 109). In DCs, QS-21 in turn triggers a MyD88-dependent inflammasome activation that involves a partial activation of the high-mobility group protein B1-TLR4 (109) and cathepsin B (shown for QS-21 in AS01), an inflammasome-dependent release of IL-1β, and the activation of both CD4^+^ and CD8^+^ T cells, and production of antibodies ((109) and reviewed in (110)). While QS-21 did not bind to either TLR2 or TLR4 (111), both lysosomal destabilization and spleen tyrosine kinase (Syk) activation were shown to be essential for both QS-21-mediated activation of monocyte-derived DCs (108), antigen cleavage, and subsequent presentation to CD8^+^ T cells (81)

However, there are several drawbacks that limit the application of QS-21, including (I) strong side effects when using high-dosages (e.g. local erythema, induration, and flu-like symptoms); (II) its chemical instability, especially in warm temperatures and pH values higher than 7.4; and (III) limited supplies due to difficulties extracting highly pure QS-21 from its natural source (107).

Therefore, in current clinical usage, QS-21 needs to be co-applied with other adjuvants and is most commonly applied packaged in liposomes in combination with MPLA (a TLR4-ligand, see above) as AS01. A study from Welsby et al. compared the effects of either QS-21 or MPLA on human DCs in culture, which were both packaged independently of each other in liposomes (108). Their results showed, that both QS-21- and MPLA-liposomes could induce higher IL-6, TNF-α, and IL-8 secretion, as well as CD86 expression (108). Interestingly these two liposome-packaged adjuvants showed different kinetics on cytokine production: MPLA-liposomes induced fast DC activation (two to four hours post-stimulation) while for QS-21-liposomes DC activation was observed at later time points (six hours post-stimulation) (108). Using microarray analysis the authors found, that QS-21- and MPLA-liposomes activated similar pathways such as cytokine-, NLR, and G-protein-couple receptor-signaling in human DCs (108). In contrast, the activation of the activator protein 1 (AP-1) and activating transcription factor 2 (ATF2)-transcription factor networks were found to be specific for QS-21-liposomes, while MPLA-liposomes specifically induced TLR- and IL-1-signaling in human DCs (108). The observed DC-activation by QS-21-liposomes was shown to depend on cholesterol-mediated endocytosis followed by lysosomal destabilization (108), which was different from MPLA since it is known to activate TLR4-signaling (60).

Although QS-21 is a strong adjuvant, it is well tolerated in clinical trials with systemic side effects being infrequent: Compared to placebo, QS-21 increased injection site pain and occurrence of diarrhea in two Pfizer-sponsored Alzheimer studies (NCT00479557 and NCT00498602) (meta-analysis in (112)). QS-21-mediated injection site pain was also reported in three phase I trials by Waite and colleagues (113) and upon intra-muscular application of QS-21 mixed with HIV-1 glycoprotein 120 (114). Also Gilewski reported local skin reactions at the injection site (duration 4 to 5 days) and mild flu-like symptoms (duration 1 to 2 days) upon application of a QS-21-adjuvanted vaccine to prevent breast cancer (115).

Although there is currently no study testing QS-21 as an adjuvant for allergy treatment, QS-21 was shown to induce Th1-biased immune responses with high titers of IgG_2a_ and IgG_2b_ antibodies (81), making it an attractive candidate for future research.

#### 3.4.2 Glucans

**Figure 4:**
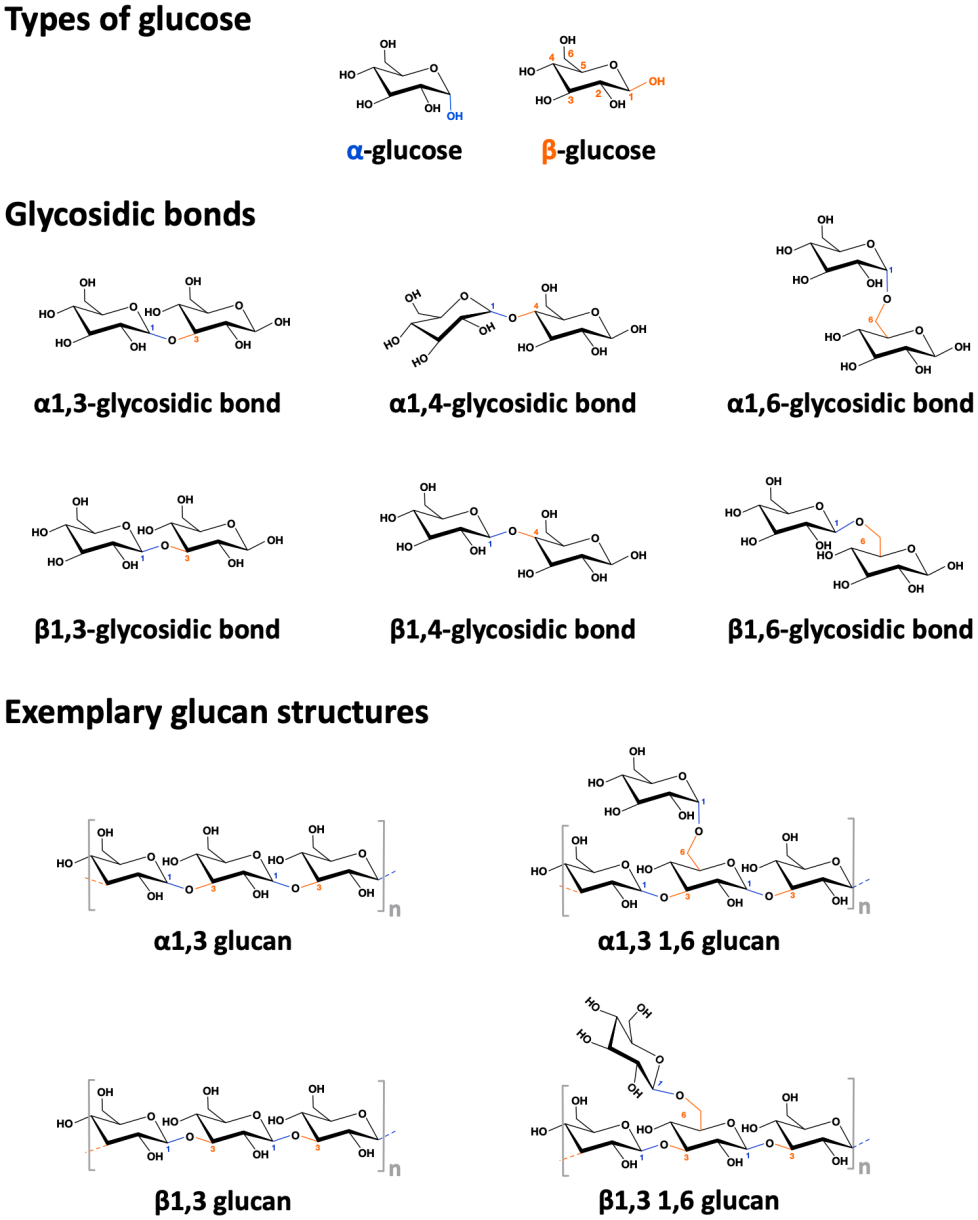
Structure of alpha- and beta-glucans. Depicted the difference between alpha- and beta-(D)-glucose, the different types of glycosidic bonds, and some simplified exemplary alpha- and beta-glucan structures.

Glucans are polysaccharides derived from either plants or microorganisms (such as bacteria, algae, or fungi) that are composed of D-glucose units linked by different glycosidic bonds (**Figure 4**) (116). Glucans encompass several different α-glucans (e.g., dextran, glycogen, pullulan, and starch) and β-glucans (e.g., cellulose, curdlan, laminarin, chrysolaminarin, lentinan, lichenin, pleuran, and zymosan) (116). The different glucan families considerably vary in structure, type of linkage, and length of the macromolecules. The mechanisms by which glucans activate immune cells are not fully understood. However, some glucans were reported to bind to either carbohydrate receptors or TLRs expressed on the surface of APCs, suggesting these molecules to have adjuvant potential (116). For example, Dillon et al. demonstrated that yeast zymosan could bind to both the Dectin-1 receptor and TLR2 on human and murine DCs, which induced higher secretion of IL-12(p70), IL-6, and IL-10 *in vitro* (117). Moreover, two studies used β-glucans chemically conjugated with either the detoxified diphtheria toxin CRM197 or the cancer-associated mucus protein mucin 1 as novel vaccines against either diphtheria or cancer (118, 119). Their results showed that, as adjuvants, β-glucans can serve as both carriers and immune activators promoting immune responses *in vitro* and *in vivo*, which enhanced the delivery of antigens by binding to APCs and enforced antigen-specific antibody production (118, 119).

The potential of glucans for allergen-specific immunotherapy is still controversial. Some researchers demonstrated β-(1,3)-glucans, which can be found in either HDM feces or pollen grains derived from several plant species, to exacerbate allergic inflammation by activating DCs *in vitro* and enhancing allergen-specific IgE antibody as well as Th2 cytokine production *in vivo* (120, 121). However, in another *in vivo* OVA-based food allergy mouse model, the authors applied either the low-molecular-weight β-(1,3)- or a 50–80% branched β-(1,6)-glucan (purified from *Aureobasidium pullulans*). Here, these two β-glucans were shown to suppress OVA-specific IgE production (122). In a non-industry-sponsored, multicenter open split-body study enrolling 105 patients with atopic dermatitis, regular topical application of a 0.25% β-glucan-based cream (Imunoglukan P4H^®^ cream) over a period of 24 weeks reduced pruritus and severity of atopic dermatitis while increasing the number of days without exacerbation (123). The β-glucan-containing cream was well tolerated with only mild local side effects (contact dermatitis and other undisclosed skin side effects) that disappeared during regular application (123). In a randomized, double-blind, parallel group, placebo-controlled study without industry sponsorship 26 patients with seasonal AR sensitized to olive pollen were treated orally with 10 mg β-(1,3–1,6)-glucan twice a day for 12 weeks (124). β-glucan treatment significantly reduced levels of Th2 cytokines and eosinophils in nasal lavage fluid while increasing levels of IL-12 (124). Recently, a β-(1,3)-glucan and a β-(1,3-1,6)-glucan both derived from *Saccharomyces cerevisiae* were investigated in an OVA-based mouse intestinal allergy model: Here, oral treatment with β-(1,3)-glucan (but not β-(1,3-1,6)-glucan) was shown to further promote intestinal inflammation and allergic responses (125), suggesting that this β-(1,3)-glucan might not be suitable for immunotherapy purposes. Gut microbiome analysis in the allergen-sensitized and β-(1,3)-glucan fed group showed an increased ratio of Firmicutes to Bacteroidetes, a potential indication of dysbiosis (125). In a phase IV clinical study (sponsored by the Meiji University of Oriental Medicine, Kyoto, Japan) oral-administration of superfine dispersed β-(1,3)-glucan (particle-size: 0.08 μm), instead of non-dispersed shiitake extract (particle-size: 288 μm), was shown to alleviate allergic symptoms like rhinorrhea, sneezing, nasal congestion, and itchy watery eyes otherwise induced by Japanese cedar pollen in allergic patients (21). Along with the reduction of the aforementioned clinical symptoms, the superfine dispersed β-(1,3)-glucan treatment group also displayed lower pollen-specific IgE antibody levels (21). Based on these findings the authors speculated, that differences in size, structure, and purification method of carbohydrates derived from natural sources might lead to distinct immune responses (21, 122). Therefore, more detailed studies are needed to understand the adjuvant potential and potential side effects of glucans.

#### 3.4.3 Mannan

Mannan is a β-(1,4)-mannose polysaccharide which can be found in the cell walls of both plant and fungal cells (81). Mannan can modulate immune responses towards both Th1 and Th2 responses (depending on its oxidation status, reduced mannan: Th2, oxidized mannan: Th1) by binding to both mannan- and C-type lectin receptors (DC-SIGN) expressed e.g., by APCs (116), which leads to activation of complement pathways (116), the NLRP3 inflammasome, as well as increased phagocytosis and cytokine secretion in APCs (81, 116). The first study using mannan as a vaccine adjuvant in mice was reported in 1992. Okawa et al. conjugated mannan with the HBV 139-147 peptide (126). The mannan:HBV peptide conjugate was shown to induce higher IgG titers in mice than corresponding dextran:HBV peptide-conjugates (126). So far, either native, oxidized, or reduced forms of mannan have been conjugated to different antigens to be tested as adjuvants for targeting APCs (reviewed in (81)). The results demonstrated both higher antigen uptake and presentation by human monocyte-derived DCs (127) coupled with the induction of distinct antigen-specific Th1- or Th2-responses in mice (128, 129).

There are some studies investigating mannan as an adjuvant for improving allergen immunotherapy. Weinberger and colleagues used oxidized mannan derived from the yeast *Saccharomyces cerevisiae* to generate reactive aldehyde groups for covalent attachment of OVA via amine-containing amino acid residues (MN–Ova) (130). Compared to OVA alone, MN–Ova was taken up more strongly by mouse DCs both *in vitro* and *in vivo*, while also inducing higher IgG antibody production in mice at the injection site (130).

**Figure 5:**
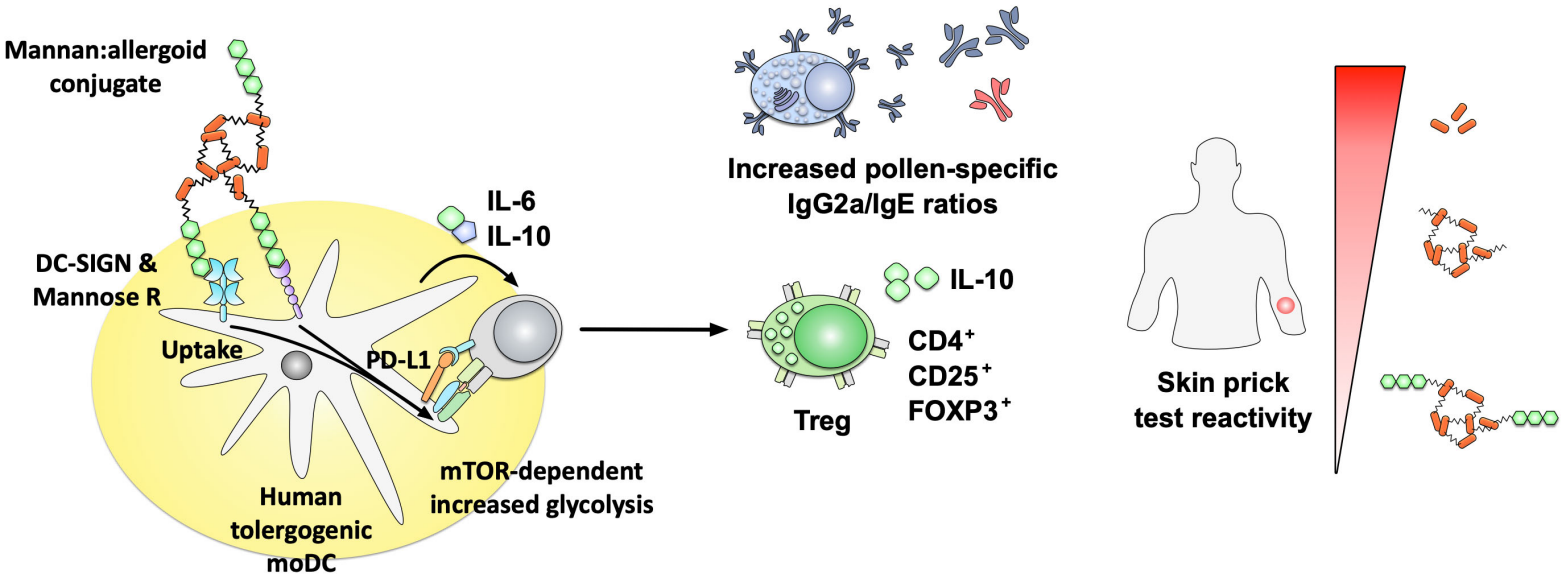
Immune modulatory properties of non-oxidized mannan:allergoid conjugates. Non-oxidized mannan:allergoid conjugates were shown to reduce skin prick test reactivity compared to either grass pollen allergens or grass pollen-based allergoids (right side). Immunologically, the mannan:allergoid conjugates taken up via DC-SIGN and mannose receptor induced higher secretion of IL-6 and IL-10 as well as surface expression of PD-L1 paralleled by an increase in mTOR-dependent glycolysis (left side). In *in vivo* analyses, mannan:allergoid conjugates stimulated the differentiation of CD4^+^CD25^high^FOXP3^+^ T_reg_ cells, paralleled by increased pollen-specific IgG_2a_/IgE ratios. In grass pollen allergic patients, the mannan allergoid conjugates induced lower skin prick test reactivity compared to either non-modified allergens or the non-conjugated allergoids alone. Results summarized according to (127). For more information see text. DC-SIGN, dendritic cell-specific intercellular adhesion molecule-3-grabbing non-integrin; FOXP3, forkhead box protein 3; Mannose R, mannose receptor; mTOR, mammalian target of rapamycin.

In a second set of studies, the native form of *Saccharomyces cerevisae* mannan was conjugated to defatted grass pollen grain-based allergoids from *Phleum pratense* (PM) (127, 131, 132). PM reduced skin prick test reactivity compared to either grass pollen allergens or grass pollen-based allergoids in a phase II trial (sponsored by Inmunotek SL, Madrid Spain)(**Figure 5**) (127). Immunologically, *in vitro* stimulation of human monocyte-derived DCs (hmoDCs) with PM induced higher secretion of IL-6 and IL-10 paralleled by a lower production of the Th2 cytokine IL-4. PM additionally promoted a mTOR-dependent increase in hmoDC glycolysis, and was more efficiently taken up compared to either pollen extracts or pollen allergoids (**Figure 5**) (127, 131, 132). *In vivo*, PM promoted tolerogenic immune responses characterized by the differentiation of CD4^+^CD25^high^FOXP3^+^ T_reg_ cells and increased pollen-specific IgG_2a_/IgE ratios in mice (**Figure 5**) (127). These immune-modulating properties were strongly dependent on the intact structure of the mannan, as conjugates containing oxidized mannan showed a strongly reduced uptake, IL-10 secretion, programmed death-ligand 1 (PD-L1) expression, and frequency of induced T_regs_ (127).

In a follow-up pre-clinical study, Benito-Villalvilla et al. could show non-oxidized mannan conjugated to allergoids to induce the differentiation of tolerogenic DCs from monocytes (133). In the presence of IL-4, GM-CSF, and the mannan:allergoid conjugates, monocytes from both non-atopic and allergic donors differentiated into tolerogenic DCs with an increased mitochondrial oxidative phosphorylation (OXPHOS) and epigenetic reprogramming characterized by an open chromatin structure with both increased histone 3 lysine 4 (H3K4) trimethylation at the promotors of IL-10, PD-L1, suppressor of cytokine signaling 3 (SOCS3), and increased histone 3 lysine 27 (H3K27) acetylation at the promotor regions of PD-L1 and SOCS1 (133). Furthermore, monocyte-derived tolerogenic DCs expressed higher amounts of the microRNAs 146 a and b while having reduced levels of miR-155 and histone deacetylases (133). These tolerogenic DCs secreted IL-6 and IL-10 and promoted the differentiation of IL-10-producing CD4^+^CD25^+^FOXP3^+^ T_regs_ (133). These results suggest mannan, besides its direct immune activating effects, to also trigger extensive epigenetic modifications in the stimulated DCs that may (if also happening in their bone marrow progenitors) potentially result in long-term modification of the respective cells reactivity towards either mannan or the conjugated antigens (so called “trained immune responses”).

In a first multicenter, prospective, randomized, double-blind, double-dummy, placebo-controlled, phase II study encompassing 196 patients with HDM allergic rhinitis with or without asthma (financed by Inmunotek SL, Madrid, Spain), patients and healthy controls were treated with a HDM extract consisting of a mixture of 50% Der p and 50% Der f allergoids conjugated to non-oxidized mannan either sublingually or subcutaneously (22). Here, both s.c. and s.l. application of the mannan:allergoid conjugates increased the percentage of patients showing an improved nasal provocation test up to 50% and reduced combined symptom medication scores by 45 to 70%, depending on the dose and application route (22). Interestingly, s.l. application of the mannan conjugates induced lower levels of HDM-specific IgG_4_ antibodies than the s.c. application route (22). Reported side effects were grade I and II reactions (mostly delayed) with all grade II reactions occurring in the 1000 mTU/ml s.c. group (but not in groups receiving higher concentrations) (22). No grade III or IV systemic reaction were reported upon application of the mannan:allergoid conjugates (22). The majority of local reactions (observed in the s.c. treatment group) were mild and occurred after the first injections. However, six delayed reactions were described as severe and led to withdrawal of the respective patients from the trial (22).

Currently, three more phase II clinical trials (EudraCT nos. 2014-005471-88, 2018-002522-23, and 2020-004126-32) as well as two phase III clinical trials for allergoid-mannan conjugates for the treatment of patients with mild to moderate HDM-induced asthma and rhinitis/rhinoconjunctivitis (NCT05400811) or birch pollen-induced allergic rhinitis/rhinoconjunctivitis (EudraCT: 2021-002252-36) are either ongoing or finished. All studies are financed by Inmunotek SL (Madrid, Spain).

In summary, these studies demonstrated the potential of mannan as an adjuvant to improve AIT, confirming its efficacy in a first clinical study with more phase II and III studies currently ongoing.

### 3.5 Toll-like receptor agonists

Innate immune cells express a highly conserved set of PRRs (e.g., TLRs and NOD-like receptors (NLRs)) that directly recognize unique structures exclusively associated with foreign microorganisms, so-called PAMPs (134). After PAMPs bind to PRRs, innate immune cells become activated, present pathogen-derived peptides to naïve T cells, and initiate and regulate adaptive immune responses (134). In this context, different PRR-ligands induce distinct activation programs in APCs, resulting in the promotion of either Th1-, Th2-, and/or Th17-immune responses (134). Due to their intrinsic immune activating potential, PRR-ligands are highly interesting adjuvant candidates for future vaccine development. Here, we will present two TLR-ligands in more detail: the TLR5-ligand flagellin, and the TLR9-ligands CpG oligodeoxynucleotides, which have been studied for their potential to improve allergen immunotherapy.

#### 3.5.1 Flagellin

The TLR5- and NLR family caspase activation and recruitment domain (CARD) containing 4 protein (NLRC4)-ligand flagellin is a bacterial motility protein forming the main body of the bacterial flagellum (135). Vaccines adjuvanted with flagellin were reported to be both safe and well-tolerated in clinical trials (136, 137) where flagellin was demonstrated to be an effective mucosal adjuvant triggering Th1-biased, protective immune responses in mice and monkeys (138–140).

Flagellin has also been analyzed pre-clinically as an adjuvant for improving traditional AIT. The available literature is divided into investigating (I) the mixture of flagellin and different allergens or (II) flagellin fused to allergens as part of fusion proteins. In this context, flagellin-containing fusion proteins were suggested as potential therapeutics combining the adjuvant activity of the TLR5- and NLRC4-ligand and the cargo antigen into a single molecule, allowing for the efficient targeting of antigens to, and simultaneous activation of TLR5^+^ APCs (140–143).

Lee et al. tested either intranasal or intralymphatic injection of *Vibrio vulnificus* flagellin B (FlaB) mixed with OVA in an OVA-based mouse model of airway inflammation (144, 145) where the OVA/FlaB mixture reduced OVA-induced airway hyper-responsiveness, inflammatory cell infiltration in lung tissue, as well as both systemic (IL-4, IL-5, IL-6, IL-17, and IFN-γ) and local (IL-4 and IL-5) cytokine production; while increasing OVA-specific IgA levels in serum (144, 145). Mechanistically, the ability of the OVA/FlaB mixture to suppress allergic responses was found to be dependent on TLR5 (146). Moreover, FlaB promoted the differentiation of IL-10- and TGF-β-producing tolerogenic DCs (tolDCs), which suppressed both Th1/Th2 responses and enhanced the activation of T_reg_ cells (146). Besides DCs and T cells, a recent study demonstrated flagellin to stabilize OVA-induced eosinophil activation (releasing major basic protein (MBP) and peroxidase (EPX) *in vitro* and *in vivo*) and to reduce oxidative stress in OVA-sensitized eosinophils which was correlated with decreased allergic inflammation (147). Additionally, Zeng et al. found flagellin to diminish allergen-induced oxidative stress in B_regs_ from both a food allergy (FA) mouse model and patients’ blood samples (148). Here, adding flagellin to OVA improved traditional AIT in an OVA-induced food allergy model *in vivo* (148). These results showed that flagellin can activate different cell types, changing both their cytokine secretion and metabolic status, which correlated with improved treatment outcomes in AIT in mice.

On the other hand, flagellin:allergen fusion proteins have demonstrated an even higher potential to improve allergy treatment than the mixture of flagellin and allergen. Tan et al. generated a recombinant fusion protein consisting of FlaB fused to the C-terminus of the HDM allergen Der p 2 (rDerp2:FlaB) (149). Compared to the non-fused Der p 2 + FlaB mixture, rDerp2:FlaB more efficiently suppressed airway hyper-responsiveness, serum IgE levels, and secretion of Th2 cytokines into bronchoalveolar lavage fluid in a HDM-induced mouse asthma model (149).

Moreover, Kitzmüller and colleagues also demonstrated that *Salmonella* FliC (genetically modified to lack the middle hypervariable domain of flagellin described to induce neutralizing anti-flagellin antibody responses) fused to either the N- or C-terminus of Bet v 1 (rFliC:Betv1 or rBetv1:FliC, respectively) could activate monocyte-derived DCs from pollen-allergic patients (150). Immunization of mice with either rFliC:Betv1 or rBetv1:FliC fusion proteins induced lower serum levels of Bet v 1-specific IgE paralleled by a higher production of IgG antibodies (150), suggesting flagellin:allergen fusion proteins to be promising novel treatment options for pollen allergy.

In own previous studies we generated fusion proteins combining flagellin A (FlaA) from *Listeria monocytogenes* with different allergens (141–143, 151). We demonstrated that prophylactic vaccination with rFlaA:OVA, but not the mixture of both proteins, induced higher production of allergen-specific IgG_2a_ antibodies paralleled with decreased levels of both IgE and Th2 cytokines (IL-5 and IL-13), resulting in the prevention of clinical symptoms such as softness of faces, body weight loss, and drop in core body temperature in an OVA-induced intestinal allergy mouse model (142, 152).

**Figure 6:**
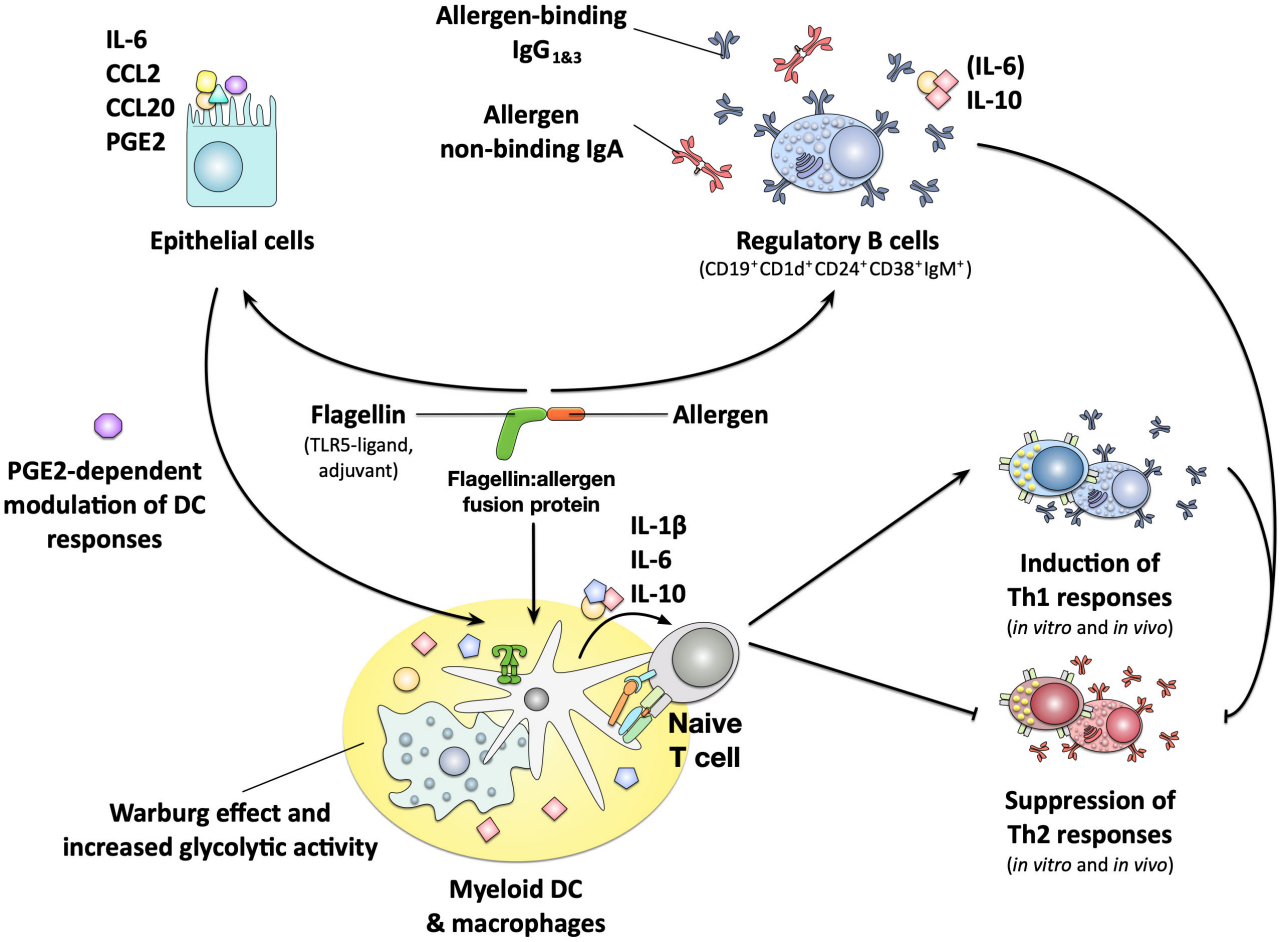
Immune modulating properties of flagellin:allergen fusion proteins. Flagellin:allergen fusion proteins combining the TLR5- and NLRC4-ligand flagellin and different allergens into a single molecule were shown to strongly activate both myeloid DCs and macrophages, resulting in the secretion of both pro- and anti-inflammatory cytokines and the expression of co-stimulatory molecules. The activated APCs promoted the induction of Th1 responses both *in vitro* and *in vivo* while also efficiently suppressing Th2 responses in an IL-10-dependent manner. This pro-tolerogenic phenotype of the APCs was critically dependent on a metabolic shift towards increased glycolytic activity termed the Warburg effect. The fusion protein consisting of flagellin A and the major birch pollen allergen Bet v 1 was also shown to strongly activate epithelial cells, resulting in the secretion of IL-6 and the chemokines CCL2 and CCL20. The activated epithelial cells furthermore produced prostaglandin E2 (PGE2) which was shown to modulate DC responses towards the fusion protein. Finally, B cells stimulated with the fusion protein were shown to form a CD19^+^CD1d^+^CD24^+^CD38^+^IgM^+^ subpopulation with regulatory properties that secreted anti-inflammatory IL-10, produced allergen-binding IgG_1&3_ antibodies and suppressed allergen-specific Th2 responses. For more information see text. PGE2, Prostaglandin E2.

More in-depth analyses showed, that all generated rFlaA:allergen fusion proteins stimulated mDCs to secrete higher amounts of both pro- (IL-6, IL-1β, TNF-α, and IL-12) and anti-inflammatory (IL-10) cytokines (**Figure 6**) (141, 143, 151–153). rFlaA:allergen fusion protein-treated mDCs also displayed an activated phenotype with higher surface expression levels of the maturation markers CD40, CD69, CD80, and CD86 (**Figure 6**) (141, 151). Mechanistically, the induced mDC activation was mediated by intracellular MyD88-, MAPK-, and nuclear factor kappa-light-chain-enhancer of activated B cells (NFκB)-pathways while being largely TLR5-independent (143, 152, 153). The rFlaA:allergen fusion protein-stimulated mDCs efficiently modulated allergen-specific T cell responses, suppressing allergen-induced Th2 cytokine (IL-4, IL-5, and IL-13) production from *ex vivo*-isolated, Th2-biased CD4^+^ T cells in an IL-10-dependent manner (**Figure 6**) (142, 143, 151). The flagellin:antigen fusion proteins formed high-molecular aggregates possibly due to self-assembly of flagellin molecules into flagella-like structures, enabling the formation of intermolecular cysteine bridges between the fused allergens (FlaA itself does not contain cysteine residues) (143, 152). These aggregates were both taken up more strongly by mDCs and inhibition of aggregate uptake dose-dependently suppressed fusion protein-induced cytokine secretion (143). Therefore, these results suggest the strong activation of mDCs by the flagellin:allergen fusion proteins, while being mostly TLR5-independent, to be at least in part caused by aggregation-mediated enhanced uptake and subsequent triggering of above-described intracellular signaling cascades.

Interestingly, the tolerogenic phenotype of mDCs stimulated with a flagellin fusion protein incorporating the major birch pollen allergen Bet v 1 (rFlaA:Betv1) was shown to depend on a JNK-MAPK-dependent activation of the mTOR pathway (143, 153). mTOR is a conserved serine/threonine protein kinase that belongs to the phosphatidylinositol 3-kinase-(PI3K) family. mTOR not only integrates various nutritional and environmental stimuli, including levels of growth factors, cellular energy reserves, and stress levels, but is also a master regulator of cellular metabolism that affects innate and adaptive immune responses (154). mTOR activation in rFlaA:Betv1-stimulated mDCs resulted in alterations of mDC metabolism with increased rates of glycolysis, Warburg metabolism, and reduced mitochondrial respiration (155). Inhibition of either glycolysis or mTOR activation suppressed rFlaA:Betv1-induced IL-10 secretion (143, 153, 155). In addition to glycolysis, fatty acid synthesis also significantly contributed to rFlaA:Betv1-mediated cytokine secretion, the production of antimicrobial molecules, and the modulation of T cell responses (155).

In line with the results presented for mDCs, stimulation of murine bone marrow-derived macrophages with rFlaA:Betv1 also triggered a MyD88-dependent, but only partly TLR5-dependent, cytokine production, stronger activation of hypoxia inducible factor 1 alpha (HIF-1a)-, MAPK-, NFκB-, and mTOR-signaling, and increased glucose metabolism (156). Therefore, macrophages also contribute to the strong immune-modulating effects of rFlaA:Betv1 previously observed *in vivo* and should be considered important target cells contributing to the re-establishment of allergen tolerance in AIT.

Moreover, the murine lung epithelial cell line LA-4 was shown to be activated by rFlaA:Betv1. Here, the fusion protein was shown to be taken up more strongly, triggering a MAPK-, NFκB-, and cyclo oxygenase 2 (COX2)-dependent activation of epithelial cells, characterized by a pronounced secretion of the cytokine IL-6, the myeloid chemo-attractants CCL2 and CCL20, as well as production of prostaglandin E2 (**Figure 6**) (157). rFlaA:Betv1-stimulated LA-4 cells modulated the activation of mDCs in a COX-2-dependent manner, resulting in lower secretion of the pro-inflammatory cytokines IL-12 and TNF-α, while the levels of the IL-1β and IL-10 remained unchanged (157). Therefore, epithelial cells also contribute to the immune modulating properties of potential allergen therapeutics and should be kept in mind as potential target cells for AIT approaches.

Finally, rFlaA:Betv1 was shown to induce a MyD88- and mTOR-dependent, but thymus-independent activation of *ex vivo*-isolated B cells, inducing a regulatory B cell phenotype (CD19^+^CD1d^+^CD24^+^CD38^+^IgM^+^) characterized by: (I) the secretion of the anti-inflammatory cytokine IL-10, (II) the production of antigen-binding IgG(1&3) and non-antigen-binding IgA antibodies, and (III) the capacity to suppress Bet v 1-induced Th2-responses (**Figure 6**) (158). To our knowledge, this is the first report showing the induction of B_regs_ by a potential therapeutic candidate for the treatment of allergic diseases.

So far Flagellin:antigen fusion proteins have not been investigated clinically for allergy treatment, but they were tested in several phase II clinical trials for the prevention of influenza infection (136, 137, 159–161). In these studies, the vaccines were well tolerated with lower doses (up to 3 µg) resulting in mild side effects (injection site pain, headache, fatigue, and myalgia) paralleled by increased serum CRP levels (136, 137, 159–161). Higher doses (10 µg) increased severity of fatigue, headache, myalgia, joint pain, fever, and gastrointestinal symptoms (nausea, vomiting, diarrhea) (136, 160). Symptoms resolved within 24 h of vaccination (136).

In summary, both flagellin and especially flagellin-containing fusion proteins incorporating different allergens were shown to efficiently activate many types of immune cells and thereby modulate immune responses, making these allergen-adjuvant conjugates interesting candidates for further development in AIT treatment.

#### 3.5.2 CpG oligodeoxynucleotides

Bacterial DNA contains a high frequency of unmethylated CpG motifs that can bind to TLR9 and trigger immune responses (162). Therefore, synthetic immunostimulatory oligodeoxynucleotides containing unmethylated CpG motifs (CpG ODN) have been investigated to either mimic natural bacterial infection or as vaccine adjuvants (162). So far, several different types of CpG ODNs have been tested in clinical phase I/II trials as vaccine adjuvants against either infectious diseases (hepatitis B, malaria, and pneumonia) or cancer (melanoma, breast cancer, sarcoma, ovarian cancer, glioblastoma, and lymphoma) (162). The overall results showed that CpG ODN adjuvants only induced short-term mild-to-moderate adverse effects, while inducing Th1-biased immune responses and promoting CD8^+^ T cell activation (162).

**Figure 7:**
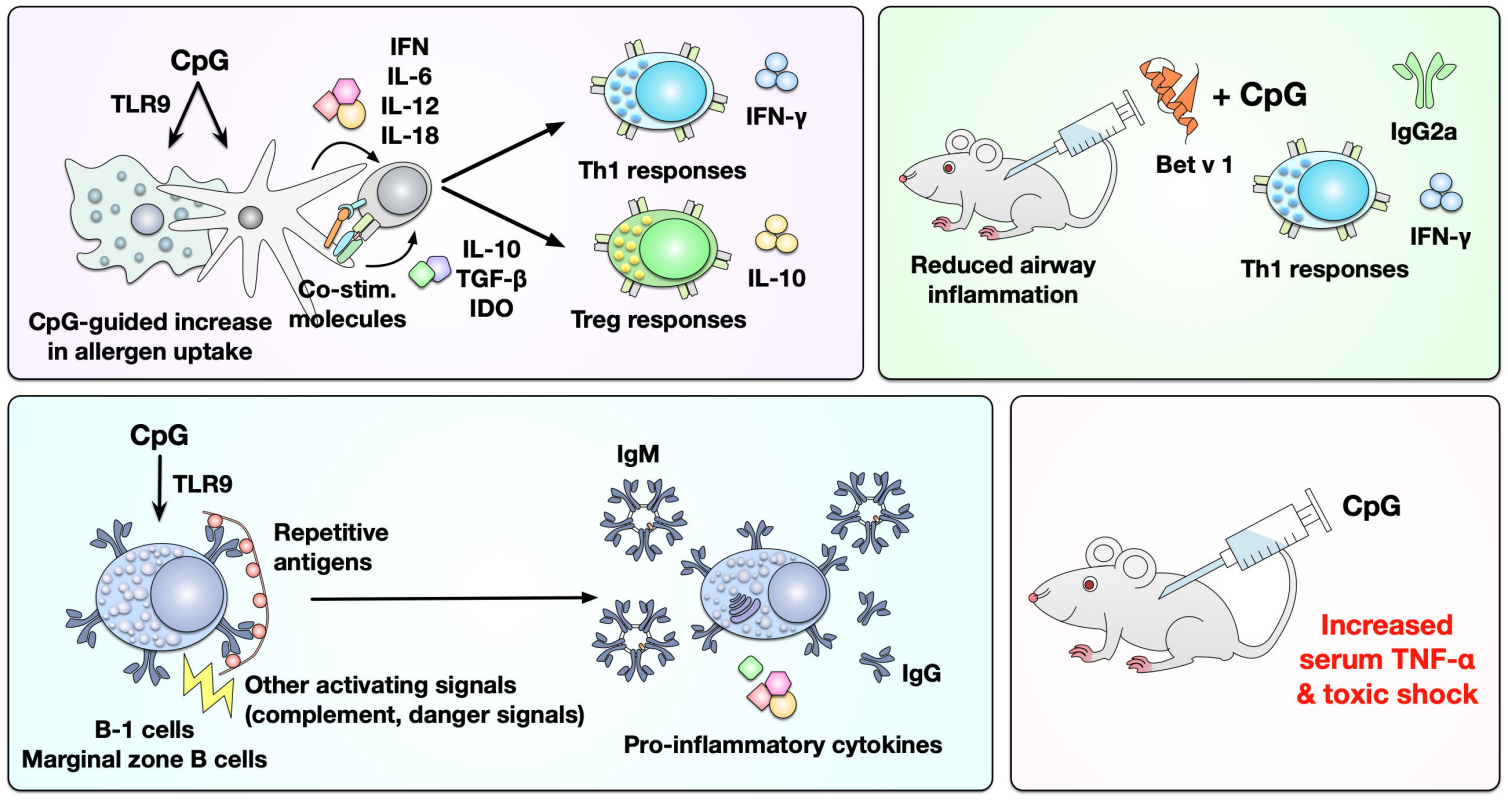
Immunological effects of CpG ODNs as adjuvants in mice and men. CpG ODNs can activate DCs and macrophages via TLR9, resulting in increased uptake of co-applied allergens and the differentiation of both allergen-specific Th1 and T_reg_ cells. In mice the mixture of Bet v 1 and CpG ODN was shown to predominantly induce IFN-γ secreting Th1 cells and the production of Bet v 1-specific IgG_2a_ antibodies. Furthermore, CpG ODNs can thymus-independently activate either B-1 or marginal zone B cells, promoting the secretion of antibodies and pro-inflammatory cytokines. Likely because of this strong capacity of CpG ODNs to activate multiple immune cells they were also reported to increase serum levels of TNF-α that were associated with toxic shock in mice. For more information see text. IDO, indoleamine 2,3-dioxygenase.

The proposed advantages of using CpG ODN for allergen-specific immunotherapy include: (I) CpG ODN can activate DCs and macrophages via TLR9, promoting especially pDCs, to increase allergen uptake and produce IL-10, TGF-β, and indoleamine 2,3-dioxygenase (IDO) (**Figure 7**). This in turn drives differentiation and activation of T_reg_ responses while also inducing DC-derived secretion of IFN, IL-6, IL-12, and IL-18 that promote the differentiation of IFN-γ producing Th1 cells and isotype-switch towards IgG_2a_ (**Figure 7**) (162). (II) Since B cells express high levels of TLR9, CpG ODN can also directly activate B cells (**Figure 7**). An *in vivo* study that used ragweed pollen-sensitized mice demonstrated that combining CpG ODN with ragweed pollen could induce higher frequencies of IL-10-producing B_reg_ (B220^+^CD19^+^CD23^+^IgM^+^CD40^+^MHCII^hi^), which contribute to the suppression of allergen-induced inflammatory responses (163). However, CpG ODN application was also reported to result in increased serum levels of TNF-α that were associated with toxic shock in mice (**Figure 7**) (164, 165).

In a ragweed allergen-induced mouse asthma model, CpG ODN was shown to induce IFN-γ-dependent Th1-responses while suppressing allergic lung inflammation (166). As an approach to treat ragweed allergy, the immune-stimulating sequence 1018 (ISS-1018, 5’-TGACTGTGAACGTTCGAGATGA-3’) was chemically conjugated to the major ragweed allergen Amb a 1, resulting in an Amb a 1:CpG conjugate with a mean conjugation rate of 1 to 4 (167). This conjugate was termed TOLAMBA. In BALB/c mice, TOLAMBA induced Th1-biased immune responses characterized by the production of IFN-γ, IgG_1_- (also observed in rabbits and cynomolgus monkeys) and IgG_2a_-antibodies, while the non-conjugated mixture of Amb a 1 and ISS 1018 predominantly induced Th2-biased immune responses (167). In Amb a 1-sensitized and -challenged BALB/c mice, TOLAMBA reduced the production of IgG_1_ and IgE antibodies while boosting production of IgG_2a_ (167). Similar results were described for Amb a 1-ISS-1080 by both Marshall et al. and Santeliz et al. (168, 169) as well as OVA-CpG ODN (1826) by Shirota et al. (170).

In 2006 Creticos and coworkers reported the results of a randomized, double-blind, placebo-controlled phase II trial testing TOLAMBA in 25 ragweed allergic patients (sponsored by the Johns Hopkins University School of Medicine, Baltimore, USA under participation of Dynavax Technologies, Berkeley, California, USA) (171). Patients were treated with six weekly injections of increasing doses of TOLAMBA (0.06, 0.3, 1.2, 3, 6, and 12 µg) and monitored during the following two ragweed seasons (171). Compared to placebo, TOLAMBA reduced rhinitis visual analogue scores by two thirds in both ragweed seasons, and also Amb a 1-specific IgE levels were lower during the first ragweed season (171). While the authors reported no vaccine-associated systemic adverse events or clinically significant abnormalities, also no effect on the primary endpoint (nasal vascular permeability as assessed by nasal-lavage albumin) was achieved (171).

To follow up on these results, a phase IIb study with TOLAMBA in 738 ragweed allergic patients was conducted (sponsored by Dynavax Technologies). It was initially reported in a conference abstract, that treatment during the first ragweed season was well tolerated in all groups without TOLAMBA-related serious adverse events (172). However, later on, the company reported problems with the study as they observed no measurable disease in any of the study groups during the ragweed season (http://investors.dynavax.com/releasedetail.cfm?releaseid=231013). In May 2008 the company reported, that although TOLAMBA showed a trend towards a reduction of the symptoms of ragweed allergic individuals relative to placebo, no statistical significance was achieved (https://investors.dynavax.com/news-releases/news-release-details/dynavax-tolambatm-chamber-study-misses-primary-endpoint-company). With no original paper published on the study so far, the company referred to “an unexpectedly high degree of variability in the data set possibly due to the subjective nature of symptom scoring used to assess efficacy” and decided to discontinue clinical development of TOLAMBA (https://investors.dynavax.com/news-releases/news-release-details/dynavax-tolambatm-chamber-study-misses-primary-endpoint-company).

As an approach to treat food allergy, Rodriguez et al. treated BALB/c mice sensitized to the major peach allergen Pru p 3 with a mixture of Pru p 3 peptides and CpG ODNs (173). In their experimental model, treatment with Pru p 3 peptides plus CpG ODNs suppressed the temperature drop induced by i.p. challenge with the allergen and strongly reduced Pru p 3-specific IgE levels (173). This CpG ODN-mediated immune modulation was accompanied by an increased production of Pru p 3-specific IgG_2a_ antibodies as well as an increase in IL-10-producing CD4^+^CD25^+^ T_regs_ and IFN-γ-producing Th1 cells (173). Interestingly, the tolerance was maintained even after stopping the treatment for three weeks (173).

In a recent review article Montamat and colleagues recapitulated the development of CpG ODN adjuvants for allergy treatment in more detail (174). When analyzing the complex immune-stimulating properties of CpG ODN adjuvants, they concluded that the detrimental inflammation and Th1 responses observed upon application of CpG ODN adjuvants may be either preferentially triggered by low doses of CpG ODNs or be the byproduct of LPS potentially contaminating the used CPG ODN preparations (174). They concluded, that application of higher doses of CpG ODN may reliably induce the desired, pro-tolerogenic T_reg_ responses (174). They proposed, that “doses between 0.5 and 1.5 mg/kg, dependent on the administration route, should induce the desired immune tolerance toward the allergen with a minimal risk of adverse reactions” (174).

In theory, the immune-activating properties of CpG ODNs are of interest in allergy treatment and initial mouse studies showed promising immune-modulating effects [induction of Th1 responses paralleled by suppression of Th2 responses (167)]. However, currently there are no clinical trials demonstrating a clear benefit of CpG ODNs in allergic patients. As neither an optimal dose nor application route of CpG ODNs are established, further studies improving on the published protocols should investigate the potential of CpG ODNs as adjuvants for advanced AIT treatment.

## 4 Summary and conclusion

Adjuvants have the potential to improve AIT by increasing the immunogenicity of isolated allergen(s), while at the same time reducing the number of injections and (cumulative) amounts of allergen(s) that need to be applied. Furthermore, adjuvants can reduce the probability of anaphylactic reactions by adsorbing the allergen(s) and modulate allergen-specific immune responses towards either predominantly tolerogenic or Th1-biassed immune responses.

The adjuvants currently approved for use in humans (aluminum hydroxide, calcium phosphate, MCT, and MPLA) show advantages for improving AIT, such as prolonging the contact time of immune cells with the allergen(s) at the injection site and inducing allergen-specific IgG production. However, some of them also show disadvantages, for example, the use of aluminum hydroxide can induce unwanted Th2 responses and IgE production.

Therefore, several different adjuvants (liposomes, O/W emulsions, VLPs, viral components, carbohydrate-based adjuvants, flagellin, and CpG ODN) have been tested in recent years not only *in vitro* and *in vivo* in respective animal models, but also in clinical trials. Liposomes, while usually lacking pronounced intrinsic immune-activating capacity, can both reduce cutaneous anaphylactic reactions and lung inflammation in mouse models when combined with other adjuvants (e.g. with QS-21 and MPLA in AS01). Similarly, O/W emulsions reduce the chance of anaphylactic reactions by slowly releasing the allergen, while generating mixed Th1/Th2 responses by increased APC recruitment and antigen uptake. So far, O/W emulsions have not seen widespread investigation for allergy treatment. VLPs are potent activators of DCs and B cells that have repeatedly shown promising results in pre-clinical trials. However, the only candidate so far tested in multiple clinical trials (QβG10) did not progress in clinical development after failing to show significant differences between the QβG10 treatment and placebo groups in a phase IIb study. However, there are currently several VLP-based therapeutics in different stages of pre-clinical and clinical development. Carbohydrate-based adjuvants like QS-21, glucans, and mannan induce a robust immune system activation with a tendency towards Th1-biassed immune responses. Here, especially mannan:allergoid conjugates have shown promising tolerogenic properties and are currently undergoing clinical evaluation in multiple trials. The TLR5-ligand flagellin fused to different allergens has shown promising pre-clinical results inducing the differentiation of tolerogenic DCs and macrophages and regulatory B cells. However, so far flagellin-containing therapeutics have not progressed to clinical trials in the field of allergology. Finally, TLR9-activating CpG oligodeoxynucleotides have shown promising immune-modulating effects in mice (induction of Th1 responses paralleled by suppression of Th2 responses), but so far there are no clinical trials demonstrating a clear benefit in allergic patients.

In summary, these adjuvants showed the potential to improve AIT by (I) inducing higher levels of allergen-specific IgG while decreasing IgE production and (II) promoting the differentiation and activation of different regulatory immune cells, such as T_regs_, tolerogenic DCs, macrophages, and B_regs_. In the performed clinical studies, some adjuvants failed to show clinical efficacy (e.g., CpG, QβG10, **Table 1**), while some adjuvants have recently shown promising results in first clinical studies and could be useful as future adjuvants (e.g., VLPs displaying allergens, PreS, superfine dispersed β-(1,3)-glucan, mannan, **Table 1**). However, up to now, these results have not resulted in marketing authorization of novel adjuvanted AIT products. With a number of clinical studies currently ongoing to further test some of the described adjuvants, we may be able to better assess the potential of some of these adjuvants in the near future.

## Author contributions

Y-JL: Data curation, Investigation, Visualization, Writing – original draft, Writing – review & editing. JZ: Data curation, Investigation, Writing – original draft, Writing – review & editing. SS: Conceptualization, Data curation, Project administration, Supervision, Validation, Visualization, Writing – original draft, Writing – review & editing.

## Glossary

**Table:**

AIT	allergen-specific immunotherapy
alum	aluminum hydroxide
AP-1	activator protein 1
APCs	antigen-presenting cells
AR	allergic rhinitis
AS01/03	adjuvant system 01/03
ATF2	activating transcription factor 2
CARD	caspase activation and recruitment domain
COX2	cyclo oxygenase 2
CpG ODN	CpG oligodeoxynucleotides
CRM197	detoxified diphtheria toxin
CuMV	cucumber mosaic virus
CYT003	VLPs derived from the bacteriophage Q beta
DCs	dendritic cells
DC-SIGN	dendritic cell-specific ICAM-3-grabbing non-integrin
DAMPs	damage-associated molecular patterns
EPX	peroxidase
FA	food allergy
FDA	United States Food & Drug Administration
FlaA	flagellin A from *Listeria monocytogenes;*
FlaB	flagellin B from *Vibrio vulnificus;*
H3K4/27	histone 3 lysine 4/27
HBV	hepatitis B virus
HCRT	hypocretin
HDM	house dust mite
HIF-1a	hypoxia inducible factor 1 alpha
HIV(-1)	human immunodeficiency virus type (1)
HIV-TAT	HIV trans-activator of transcription
hmoDCs	human monocyte-derived DCs
HPV	human papillomavirus
IDO	indoleamine 2,3-dioxygenase
i.v.	intravenous
i.p.	intraperitoneal
ISS-1018	immune-stimulating sequence 1018
JNK	c-Jun N-terminal kinase
LPS	lipopolysaccharide
LRR	leucine rich repeats
MAPK	mitogen activated protein kinase
MBP	major basic protein
MCT	microcrystalline tyrosine
mDCs	myeloid dendritic cells
MN–Ova	oxidized mannan derived from *Saccharomyces cerevisiae* conjugated to OVA
MPLA	monophosphoryl lipid A
mTOR	mammalian target of rapamycin
MyD88	myeloid differentiation primary response protein 88
NE	nanoscale oil-in-water emulsions
NF-κB	nuclear factor kappa-light-chain-enhancer of activated B cells
NLR	NOD-like receptor
NLRC4	NLR family CARD domain containing 4 protein
NLRP3	NOD-, LRR- and pyrin domain-containing protein 3
NOD	nucleotide oligomerization domain
O/W	oil-in-water
OVA	ovalbumin
OXPHOS	oxidative phosphorylation
PAMPs	pathogen-associated molecular patterns
PD-L1	programmed death-ligand 1
pDCs	plasmacytoid DCs
PGE2	prostaglandin E2
PI3K	phosphatidylinositol 3-kinase
PM	grass pollen grain-based allergoids from *Phleum pretense;* PN-NE, NE-formulated peanut allergens
PN-NE	NE-formulated peanut allergens
PQ	Pollinex Quattro^®^
PreS	T-cell epitope derived from the hepatitis B virus
PRRs	pathogen recognition receptors
QβG10	bacteriophage Q beta
QS-21	Saponin-based adjuvants
rDerp2:FlaB	flagellin B from *Vibrio vulnificus* fused to the C-terminus of the HDM allergen Der p 2
s.c.	subcutaneous
s.l.	sublingual
SCIT	subcutaneous allergy immunotherapy
SOCS3	suppressor of cytokine signaling 3
Syk	spleen tyrosine kinase
ST-SIT	short-term specific immunotherapy
TAT	trans-activating regulatory protein
TGF-β(1)	transforming growth factor beta (1)
TLR	“Toll”-like receptor
TOLAMBA	Amb a 1:CpG ODN conjugate
tolDCs	tolerogenic dendritic cells
VLP	virus-like particles
VP1	viral protein 1
